# Supplementary material for: From multiplicity of infection to force of infection in sparsely sampled high-transmission Plasmodium falciparum populations
Source: eLife. 2026 Jun 23;13:RP100076. doi: 10.7554/eLife.100076 (PMC13290237; doi:10.7554/eLife.100076)
Supplement: Peer review history

# Editor report: eLife Assessment

**DOI:** 10.7554/eLife.100076.4.sa0

**Authors:** Isabel Rodriguez-Barraquer

**Affiliations:** University of California, San Francisco United States

**Keywords:** Solid, Useful

The ability to estimate the force of infection for *Plasmodium falciparum* from other more directly measurable epidemiological quantities would contribute to malaria epidemiology. The authors propose a method to accomplish this using genetic data from the var genes of the Pf genome and novel applications of existing methods from queueing theory. After revising the manuscript, this is a **useful** contribution to the field and the authors provide **solid** evidence to support it.

# Referee report: Reviewer #2 (Public review)

**DOI:** 10.7554/eLife.100076.4.sa1

Summary:

The authors combine a clever use of historical clinical data on infection duration in immunologically naive individuals and queuing theory to infer the force of infection (FOI) from measured multiplicity of infection (MOI) in a sparsely sampled setting. They conduct extensive simulations using agent based modeling to recapitulate realistic population dynamics and successfully apply their method to recover FOI from measured MOI. They then go on to apply their method to real world data from Ghana before and after an indoor residual spraying campaign.

Strengths:

- The use of historical clinical data is very clever in this context

- The simulations are very sophisticated with respect to trying to capture realistic population dynamics

- The mathematical approach is simple and elegant, and thus easy to understand

Weaknesses:

- The assumptions of the approach are quite strong, and the authors have made clear that applicability is constrained to individuals with immune profiles that are similar to malaria naive patients with neurosyphilis. While the historical clinical data is a unique resource and likely directionally correct, it remains somewhat dubious to use the exact estimated values as inputs to other models without extensive sensitivity analysis.

Comments on revisions:

The authors have adequately responded to all comments.

# Author comment: Author response

**DOI:** 10.7554/eLife.100076.4.sa2

**Authors:** Qi Zhan, Kathryn E Tiedje, Karen P Day, Mercedes Pascual

**Affiliations:** Stanford University Stanford United States; The University of Melbourne Melbourne Australia; The University of Melbourne Melbourne Australia; New York University New York United States

The following is the authors’ response to the previous reviews

**Public Reviews:**

**Reviewer #1 (Public review):**

The authors have adequately responded to all comments.

We thank Reviewer 1 for their positive assessment of our previous round of revisions.

**Reviewer #2 (Public review):**

Summary:

The authors combine a clever use of historical clinical data on infection duration in immunologically naive individuals and queuing theory to infer the force of infection (FOI) from measured multiplicity of infection (MOI) in a sparsely sampled setting. They conduct extensive simulations using agent based modeling to recapitulate realistic population dynamics and successfully apply their method to recover FOI from measured MOI. They then go on to apply their method to real world data from Ghana before and after an indoor residual spraying campaign.

Strengths:

- The use of historical clinical data is very clever in this context

- The simulations are very sophisticated with respect to trying to capture realistic population dynamics

- The mathematical approach is simple and elegant, and thus easy to understand

Weakness:

The assumptions of the approach are quite strong, and the authors have made clear that applicability is constrained to individuals with immune profiles that are similar to malaria naive patients with neurosyphilis. While the historical clinical data is a unique resource and likely directionally correct, it remains somewhat dubious to use the exact estimated values as inputs to other models without extensive sensitivity analysis.

We thank reviewer 2 for their comments on our previous round of revisions. The statement here that “it remains somewhat dubious to use the exact estimated values as inputs to other models” suggests that we may not have been sufficiently clear on how infection duration is represented in our agent-based model (ABM) of malaria population dynamics. Because our analysis uses simulated outputs from the ABM to validate the performance of the two queuing-theory methods, we believe this point warrants clarification, which we provide below.

When simulating with the ABM, we do not use empirical estimates of infection duration in immunologically naïve individuals from the historical clinical data as direct inputs. Instead, infection duration emerges from the within-host dynamics modeled in the ABM (lines 800-816, second paragraph of the subsection Within-host dynamics in Appendix 1-Simulation data of the previous revision). Briefly, each *Plasmodium falciparum* parasite carries approximately 50-60 *var* genes, each encoding a distinct variant surface antigen expressed during the blood stage of infection. Empirical evidence[1,2] indicates that these *var* genes are expressed largely sequentially. If a host has previously encountered the antigenic product of a given *var* gene and retains immunity to it, subject to waning at empirically estimated rates[3,4], the corresponding parasite subpopulation is rapidly cleared. Conversely, if the host is naïve to that gene, it takes approximately seven days for the immune system to mount an effective antibody response, resulting in a rapid decline or elimination of the expressed variant[5]. This seven-day timescale aligns with the duration of each successive parasitemia peak observed in *Plasmodium falciparum* infections[6,7], each arising primarily from the expression of a single *var* gene and occasionally from a small number of *var* genes.

In our previous analyses, we therefore modeled an average expression duration of seven days per gene in naïve hosts. Specifically, the switching time to the next gene was drawn from an exponential distribution with a mean of seven days. Each *var* gene is represented as a linear combination of two epitopes (alleles), based on the empirical characterization of two hypervariable regions in the *var* tag region[8], and immunity is acquired against these alleles. Immunity to one allele of a given gene reduces its average expression duration by approximately half, whereas immunity to both alleles results in an immediate switch to another *var* gene within the infection. Consequently, the total duration of infection is proportional to the number of unseen alleles by the host across all *var* genes expressed during that infection (lines 800-816, second paragraph of the subsection Within-host dynamics in Appendix 1-Simulation data of the previous revision).

Prompted by the reviewer’s comments, in this revision we additionally tested mean expression durations of 7.5 and 8 days per *var* gene, together with an extension of the within-host rules. These values were applied in combination with the extended within-host rules (see the next paragraph for motivation and details). Although differences among the three mean expression durations are modest at the per-gene level, when aggregated across all *var* genes expressed within an individual parasite, the resulting total infection duration can differ by on the order of several months. The resulting distributions of infection duration across immunologically naïve individuals and those aged 1-5 years, together with those generated under our previous simulation settings, span a range of means and variances that lies above and below, but encompasses, scenarios comparable to the historical clinical data from naïve neurosyphilis patients treated with *P. falciparum* malaria. We have provided example supplementary figures illustrating that the distributions of infection duration from the simulated outputs overlap with, and closely resemble, the empirical distribution from the historical clinical data (Appendix 1-Figure 27-32).

We considered the following modification of the within-host rules. In our previous ABM simulations, we had assumed that an infection would clear only once the parasite had exhausted its entire *var* gene repertoire, that is, after every *var* gene had been expressed and recognized. However, biological evidence indicates that clearance can occur earlier for several reasons, including stochastic extinction before full repertoire exhaustion. Even if some *var* genes remain unexpressed, an infection can terminate due to demographic stochasticity once parasite densities fall to very low levels. This decline in parasite densities may result from non-variant-specific immune mechanisms or from cross-immunity among *var* genes that share sequence similarity or alleles[9,10,11], both of which can substantially reduce parasite numbers. To model the possibility of termination or clearance before full repertoire exhaustion, we implemented a simple scenario in which there is a small probability of clearing the current infection while a given *var* gene-whether non-final or final-is being expressed. This probability is a function of the host’s pre-existing immunity to the two epitopes (alleles) of that gene, thereby capturing in a parsimonious manner the effects of cross-immunity among sequence- or allele-sharing *var* genes in reducing parasitemia. Specifically, it is modeled as a Bernoulli draw whose success probability equals the immunity level against the gene (0 for no immunity to either epitope, 0.5 for immunity to one epitope, and 1 for immunity to both epitopes) multiplied by a constant factor of 0.025. Thus, the probability scales with pre-existing variant-specific immunity to the gene but remains small overall, while introducing additional variance into the emergent distribution of total infection duration across hosts.

We acknowledge that the ABM used to simulate malaria population dynamics cannot capture all mechanisms and complexities underlying within-host processes, many of which remain poorly understood. However, we emphasize that the resulting distributions of infection duration generated by the ABM span a broad range of means, variances, and shapes, including distributions that closely match those observed in the clinical historical data. Because the queueing-theory methods rely on only the mean and variance of infection duration to estimate the force of infection (FOI), these scenarios, which collectively span and encompass values comparable to the empirical ones, provide an appropriate basis for evaluating the performance of the methods using simulated outputs. We have added supplementary figures (see Appendix 1-Figure 16-22) illustrating the corresponding FOI inference results when we allow for clearance before the complete expression of the *var* repertoire, and the accuracy of FOI estimation remains comparable across all the scenarios examined.

Finally, we emphasize that the application of the queuing-theory methods to the simulated outputs and to the Ghana field survey data involve two self-contained steps. For the simulations, FOI is inferred directly from the emergent distributions of infection duration generated by the ABM. For the Ghana surveys, FOI is inferred using the historical clinical data, which remains one of the few credible and widely used empirical sources for infection duration in immunologically naïve individuals[6]. By exploring different mean expression durations and within-host rules in the ABM, which generates distributions of infection duration that span and encompass those comparable to the empirical distribution, we demonstrate that the queueing-theory methods perform comparably across diverse scenarios and are well suited for application to the Ghana field surveys.

We expanded the section on within-host dynamics in Appendix 1 to elaborate on this point (Lines 817-854).

**Reviewer #3 (Public review):**

I think the authors gave a robust but thorough response to our reviews and made some important changes to the manuscript which certainly clarify things for me.

We thank Reviewer 3 for their positive feedback on our previous round of revisions.

References

(1) Zhang, X. & Deitsch, K. W. The mystery of persistent, asymptomatic *Plasmodium falciparum* infections. Curr. Opin. Microbiol 70, 102231 (2022).

(2) Deitsch, K. W. & Dzikowski, R. Variant gene expression and antigenic variation by malaria parasites. Annu. Rev. Microbiol. 71, 625–641 (2017).

(3) Collins, W. E., Skinner, J. C. & Jeffery, G. M. Studies on the persistence of malarial antibody response. American journal of epidemiology, 87(3), 592–598 (1968).

(4) Collins, W. E., Jeffery, G. M. & Skinner, J. C. Fluorescent Antibody Studies in Human Malaria. II. Development and Persistence of Antibodies to *Plasmodium falciparum*. The American journal of tropical medicine and hygiene, 13, 256–260 (1964).

(5) Gatton, M. L., & Cheng, Q. Investigating antigenic variation and other parasite-host interactions in *Plasmodium falciparum* infections in naïve hosts. Parasitology, 128(Pt 4), 367–376 (2004).

(6) Maire, N., Smith, T., Ross, A., Owusu-Agyei, S., Dietz, K., & Molineaux, L. A model for natural immunity to asexual blood stages of *Plasmodium falciparum* malaria in endemic areas. The American journal of tropical medicine and hygiene, 75(2 Suppl), 19–31 (2006).

(7) Chen D. S., Barry A. E., Leliwa-Sytek A., Smith T-A., Peterson I., Brown S. M., et al. A Molecular Epidemiological Study of var Gene Diversity to Characterize the Reservoir of *Plasmodium falciparum* in Humans in Africa. PLoS ONE 6(2): e16629 (2011).

(8) Larremore D. B., Clauset A., & Buckee C. O. A Network Approach to Analyzing Highly Recombinant Malaria Parasite Genes. PLoS Comput Biol 9(10): e1003268 (2013).

(9) Holding T. & Recker M. Maintenance of phenotypic diversity within a set of virulence encoding genes of the malaria parasite *Plasmodium falciparum*. J. R. Soc. Interface.1220150848 (2015).

(10) Crompton, P. D., Moebius, J., Portugal, S., Waisberg, M., Hart, G., Garver, L. S., Miller, L. H., Barillas-Mury, C., & Pierce, S. K. Malaria immunity in man and mosquito: insights into unsolved mysteries of a deadly infectious disease. Annual review of immunology, 32, 157–187 (2014).

(11) Langhorne, J., Ndungu, F., Sponaas, AM. et al. Immunity to malaria: more questions than answers. Nat Immunol 9, 725–732 (2008).
